# Photonic topological phases in dispersive metamaterials

**DOI:** 10.1038/s41598-018-36170-0

**Published:** 2018-12-14

**Authors:** You-Zhong Yu, Ruey-Lin Chern

**Affiliations:** Institute of Applied Mechanics, National Taiwan University, Taipei, 106 Taiwan

## Abstract

We analyze the photonic topological phases in dispersive metamaterials which satisfy the degenerate condition at a reference frequency. The electromagnetic duality allows for the hybrid modes to be decoupled and described by the spin-orbit Hamiltonians with pseudospin 1, which result in nonzero spin Chern numbers that characterize the topological phases. In particular, the combined Hamiltonian of the hybrid modes complies with a fermionic-like pseudo time-reversal symmetry that ensures the Kramers degeneracy, leading to the topological protection of helical edge states. The transverse spin generated by the evanescent surface waves is perpendicular to the wave vector, which exhibits the spin-momentum locking as in the surface states for three-dimensional topological insulators. The topological properties of the helical edge states are further illustrated with the robust transport of a pair of counterpropagating surface waves with opposite polarization handedness at an irregular boundary of the metamaterial.

## Introduction

Inspired by the discovery of topological insulators in recent years^1–7^, there has been a surge of interest in the study of topological phases in photonic systems^8–23^. Topological insulators are insulating in the bulk but possess conducting states on their surfaces. The role of topology was first established in the study of phase transition in two-dimensional (2D) systems, known as the Kosterlitz-Thouless transition^24^. A well understood form of the topological phase was later represented by the quantum Hall (QH) state^25^, a 2D electron gas in a static magnetic field, which belongs to a topological class that breaks the time-reversal (TR) symmetry. In this system, a single edge mode, known as the *chiral* edge state, propagates unidirectionally at the boundary, which is insensitive to disorder^26^. The topological properties of the QH states are manifest on the quantized Hall conductance characterized by the TKNN invariant or Chern number^27^.

Another topological phase that preserves the TR symmetry is the quantum spin Hall (QSH) state^28–30^, in which no magnetic field is required. The spin-orbit interaction allows a different topological class when the TR symmetry is unbroken. In this system, a pair of edge modes with opposite spin, known as the *helical* edge states, counterpropagate at a given edge without backscattering^31^. The topological properties of the QSH states can be characterized by the *Z*_2_ invariant^32^ or spin Chern number^33^. The theoretical concepts developed in the QSH states were soon generalized to three-dimensional (3D) topological insulators^1,2^.

The photonic analogue of the QH states was identified in 2D gyroelectric or gyromagnetic photonic crystals^8–10^, where the gyrotropy effect breaks the TR symmetry as a static magnetic field does in the QH system. The unidirectional edge modes, which are analogous to the chiral edge states, exist in the photonic band gap with a nonzero Chern number for all bands below the gap. More recently, the photonic QH states can also be found in the Tellegen metacrystals^34^. In another aspect, the photonic QSH states were demonstrated in 2D bianisotropic photonic crystals^15,21^, where the magnetoelectric coupling emulates the effect of spin-orbit interaction. The counterpropagating edge modes, which are analogous to the helical edge states in the QSH system, exist in the frequency gap between the bulk bands with nonzero spin Chern numbers.

The helical edge states, which are doubly degenerate and TR partners of each other, form a Kramers doublet that usually exists in a TR invariant system with spin 1/2. The Kramers degeneracy, which is crucial to the emergence of helical edge states, therefore cannot readily apply to the photonic system with spin 1, unless additional symmetry has been imposed in the system. For the bianisotropic medium with the ‘spin’-degenerate condition: *ε* = *μ*^15,35–37^, the transverse magnetic (TM) and transverse electric (TE) modes propagate with identical wave numbers. The linear combinations *E*_*z*_ ± *H*_*z*_, referred to as the *pseudospin* states, become a Kramers doublet in the photonic system^15^. The spin-degenerate condition was also used in the Tellegen medium for constructing the Kramers degeneracy, where the pseudospin states are expressed as *E*_*z*_ ± *iH*_*z*_^21^. Furthermore, the hybridization of TM and TE modes was used to create the photonic topological phases in metacrystal waveguides^17,19,38–41^. The Kramers degeneracy may even exist in 2D dielectric photonic crystals^20,42,43^ without the hybridization of TM and TE modes, where the pseudospin states are represented by the combination of *p* and *d* orbital-like basis functions. In photonic systems, the Kramers degeneracy is guaranteed by the *pseudo* TR symmetry^20,21^, which can be constructed from the symmetry in crystal structure^20,44^ or constitutive relation^21^.

In the present study, we analyze the photonic topological phases in dispersive metamaterials which satisfy the degenerate condition ($\underline{\varepsilon }=-\,\underline{\mu }$) at a reference frequency *ω*_0_, around which a frequency gap between the bulk modes may exist. The electromagnetic duality allows for the hybrid modes (**E** ± *η*_0_**H**) to be decoupled at the reference frequency, which are determined by two subsystems with degenerate eigenvalues. By introducing the pseudospin states as the eigenfield basis, the hybrid modes are described by the spin-orbit Hamiltonians with pseudospin 1, which result in nonzero spin Chern numbers that characterize the topological phases. In particular, the combined Hamiltonian of the two subsystems is TR invariant under a fermionic-like pseudo TR operator *T*_*p*_ with ${T}_{p}^{2}=-\,\underline{I}$, which ensures the Kramers degeneracy of the hybrid modes, leading to the topological protection of helical edge states. The Kramers doublet consists of a pseudospin state from the plus hybrid mode (**E** + *η*_0_**H**) and another state from the minus hybrid mode (**E** − *η*_0_**H**), which are TR partners and orthogonal to each other.

For illumination, the surface modes at the interface between vacuum and the metamaterial are analytically formulated based on Maxwell’s boundary conditions, which are represented by a dispersion surface in the frequency gap and reduced to an ellipse or a circle at the reference frequency. The evanescent surface wave generates a *transverse spin* perpendicular to the wave vector, which exhibits the spin-moment locking as in the surface states for 3D topological insulators. The topological properties of the surface modes are further illustrated with the electromagnetic radiation excited by an appropriately phased point dipole at the interface. The surface waves with opposite polarization handedness counterpropagate toward different directions, which are able to bend around sharp corners without backscattering.

## Results

### Bulk modes

Consider a dispersive medium characterized by the frequency-dependent permittivity tensor ${\varepsilon }_{0}\underline{\varepsilon }(\omega )$ and permeability tensor ${\mu }_{0}\underline{\mu }(\omega )$. Treating the combined electric field *E* = (*E*_*x*_, *E*_*y*_, *E*_*z*_) and magnetic field *H* = (*H*_*x*_, *H*_*y*_, *H*_*z*_) as a six-component vector, Maxwell’s equations for the time-harmonic fields (with the convention *e*^−*iωt*^) are written in matrix form as

1 $$(\begin{array}{cc}\omega \underline{\varepsilon } & c{\bf{k}}\times \underline{I}\\ c{\bf{k}}\times \underline{I} & -\omega \underline{\mu }\end{array})(\begin{array}{c}{\bf{E}}\\ {\bf{H}}^{\prime} \end{array})=0,$$

where $\underline{I}$ is the 3 × 3 identity matrix, **H**′ = *η*_0_**H**, and ${\eta }_{0}=\sqrt{{\mu }_{0}/{\varepsilon }_{0}}$. Assume that the medium is uniaxially anisotropic with the material parameters: $\underline{\varepsilon }=\mathrm{diag}({\varepsilon }_{t},{\varepsilon }_{t},{\varepsilon }_{z})$ and $\underline{\mu }={\rm{diag}}({\mu }_{t},{\mu }_{t},{\mu }_{z})$. The existence of a nontrivial solution of **E** and **H**′ requires that the determinant of the 6 × 6 matrix in Eq. (1) be zero, which gives the characteristic equation of the bulk modes as

2 $$[{\varepsilon }_{t}({k}_{t}^{2}-{\varepsilon }_{z}{\mu }_{t}{k}_{0}^{2})+{\varepsilon }_{z}{k}_{z}^{2}][{\mu }_{t}({k}_{t}^{2}-{\varepsilon }_{t}{\mu }_{z}{k}_{0}^{2})+{\mu }_{z}{k}_{z}^{2}]=0,$$

where ${k}_{t}^{2}={k}_{x}^{2}+{k}_{y}^{2}$ and *k*_0_ = *ω*/*c*. The above bi-quadratic equation shows that the bulk modes consist of two parts with dual symmetry between *ε*_*n*_ and *μ*_*n*_ (*n* = *t*, *z*).

For analyzing the topological phases in the present medium, we assume that the medium satisfies the ‘degenerate’ condition: $\underline{\varepsilon }=-\,\underline{\mu }$ at a reference frequency *ω*_0_. In the neighborhood of *ω*_0_, *ε*_*n*_ and *μ*_*n*_ can be approximated, respectively, as ${\varepsilon }_{n}\approx {\varepsilon }_{n0}+{\frac{d{\varepsilon }_{n}}{d\omega }|}_{\omega ={\omega }_{0}}(\omega -{\omega }_{0})\equiv {\varepsilon }_{n0}+{\tilde{\varepsilon }}_{n}\delta \omega /{\omega }_{0}$ and ${\mu }_{n}\approx {\mu }_{n0}+{\frac{d{\mu }_{n}}{d\omega }|}_{\omega ={\omega }_{0}}(\omega -{\omega }_{0})\equiv {\mu }_{n0}+{\tilde{\mu }}_{n}\delta \omega /{\omega }_{0}$. If *ε*_*t*0_*ε*_*z*0_ ≠ 0 (*μ*_*t*0_*μ*_*z*0_ ≠ 0), a frequency gap between the two bulk modes exists around *ω*_0_. The band gap size can be estimated by the solutions of *ω* at *k*_*t*_ = *k*_*z*_ = 0 [cf. Eq. (2)], between which the bulk modes do not exist. For ${\varepsilon }_{n0} < 0$ (${\mu }_{n0} > 0$), the upper and lower band edges are approximated by $\omega /{\omega }_{0}\approx 1+\,{\rm{\min }}(\,-\,{\varepsilon }_{t0}/{\tilde{\varepsilon }}_{t},-{\varepsilon }_{z0}/{\tilde{\varepsilon }}_{z})$ and $\omega /{\omega }_{0}\approx 1-\,{\rm{\min }}({\mu }_{t0}/{\tilde{\mu }}_{t},{\mu }_{z0}/{\tilde{\mu }}_{z})$, respectively. Here, ${\tilde{\varepsilon }}_{t}$, ${\tilde{\varepsilon }}_{z}$, ${\tilde{\mu }}_{t}$, and ${\tilde{\mu }}_{z}$ are assumed to be positive definite^45^.

### Spin-orbit Hamiltonians

The electromagnetic duality of Maxwell’s equations dictates that the matrix in Eq. (1) is symmetric with the same diagonal elements when the degenerate condition is satisfied. This enables us to rewrite the wave equations for the hybrid modes, defined by **E** ± **H**′, at the reference frequency as

3 $$(\begin{array}{cc}{ {\mathcal H} }_{0}({\bf{k}}) & 0\\ 0 & { {\mathcal H} }_{0}(\,-\,{\bf{k}})\end{array})(\begin{array}{c}{\bf{E}}+{\bf{H}}^{\prime} \\ {\bf{E}}-{\bf{H}}^{\prime} \end{array})=0,$$

where ${ {\mathcal H} }_{0}(\,\pm \,{\bf{k}})=\omega \underline{\varepsilon }\pm c{\bf{k}}\times \underline{I}$. Note that the hybrid modes are completely decoupled and determined by two subsystems (3 × 3 matrix) with the same eigenvalues, their matrix determinants being equal: $|{ {\mathcal H} }_{0}({\bf{k}})|=|{ {\mathcal H} }_{0}(\,-\,{\bf{k}})|$. In the isotropic case, where *ε*_*t*0_ = *ε*_*z*0_ ≡ *ε* and ${\tilde{\varepsilon }}_{t}={\tilde{\varepsilon }}_{z}\equiv \tilde{\varepsilon }$, the wave equation for the plus hybrid mode **F** = **E** + **H**′ can be rearranged as (see Methods A)

4 $${ {\mathcal H} }_{+}\psi -d\psi =\delta \omega \psi ,$$

by introducing the *pseudospin* state $$\psi ={U}^{-1}\tilde{\psi }$$, where $$\tilde{\psi }={(\frac{-{F}_{x}+i{F}_{y}}{\sqrt{2}},{F}_{z},\frac{{F}_{x}+i{F}_{y}}{\sqrt{2}})}^{T}$$ and $$U={\rm{diag}}(\sqrt{{\tilde{\varepsilon }}_{z}/{\tilde{\varepsilon }}_{t}},\,1,$$$$\sqrt{{\tilde{\varepsilon }}_{z}/{\tilde{\varepsilon }}_{t}})$$. In Eq. (4), $$d={\omega }_{0}\varepsilon /\tilde{\varepsilon }$$ and

5 $${ {\mathcal H} }_{+}=\alpha {\bf{S}}\cdot \kappa ,$$

where $\alpha =c/\tilde{\varepsilon }$, $\kappa ={\kappa }_{x}\hat{x}+{\kappa }_{y}\hat{y}+{\kappa }_{z}\hat{z}=i{\bf{k}}$, ${\bf{S}}={S}_{x}\hat{x}+{S}_{y}\hat{y}+{S}_{z}\hat{z}$, and *S*_*n*_ (*n* = *x*, *y*, *z*) are the spin matrices for spin 1.

Note here that Eq. (4) is formulated as an eigensystem with *δω* as the eigenvalue. The Hamiltonian ${ {\mathcal H} }_{+}$ in Eq. (5) represents the spin-orbit interaction *S*⋅*κ* with pseudospin 1^45^, which is mathematically equivalent to the Hamiltonian of a magnetic dipole moment in a magnetic field. The imaginary wave vector *κ* in ${ {\mathcal H} }_{+}$ reflects the fact that the wave is evanescent in the frequency gap. For the minus hybrid mode **F** = **E** − **H**′ in Eq. (3), it is straightforward to show that the corresponding spin-orbit Hamiltonian is given by

6 $${ {\mathcal H} }_{-}=-\,\alpha {\bf{S}}\cdot \kappa .$$

Note that the hybrid modes can be slightly different if the corresponding Hamiltonian is rewritten in a different format^37,46,47^.

### Topological invariants

The topological properties of the spin-orbit Hamiltonians ${ {\mathcal H} }_{\pm }$ can be characterized by the topological invariants based on their eigenfields. For this purpose, we calculate the Berry flux over a closed surface *S*: ${\kappa }_{x}^{2}+{\kappa }_{y}^{2}+{\kappa }_{z}^{2}={\varepsilon }^{2}{k}_{0}^{2}$, corresponding to the bulk mode at the reference frequency *ω*_0_ in the imaginary wave vector space. The eigensystem for the plus hybrid mode in Eq. (5)

7 $${ {\mathcal H} }_{+}{\psi }_{\sigma }={\lambda }_{\sigma }{\psi }_{\sigma }.$$

is solved to give the eigenvalues *λ*_*σ*_ and eigenvectors *ψ*_*σ*_ (*σ* = ±1, 0), based on which the Chern numbers are calculated to give *C*_*σ*_ = 2*σ* (see Methods B). The nonzero topological invariants *C*_*σ*_ (*σ* = ±1) reveal the topological nature of the pseudospin states, where *σ* describes the helicity (or handedness) of the states. For this subsystem, the total Chern number $C=\sum _{\sigma }{C}_{\sigma }$ and the spin Chern number ${C}_{{\rm{spin}}}=\sum _{\sigma }\sigma {C}_{\sigma }$^33^ are given by

8 $$C=0,\,{C}_{{\rm{spin}}}=4,$$

which are consistent with the quantum spin Hall effect of light^48^. On the other hand, the eigensystem for the minus hybrid mode in Eq. (6) is given by

9 $${ {\mathcal H} }_{-}{\psi }_{-\sigma }={\lambda }_{\sigma }{\psi }_{-\sigma },$$

where the eigenvalues *λ*_*σ*_ are the same as those of ${ {\mathcal H} }_{+}$. The helicity of the eigenvectors, however, has been flipped from *σ* to −*σ*. The Chern numbers are therefore change signs as *C*_*σ*_ = −2*σ*. For this subsystem, the total and spin Chern numbers are given by

10 $$C=\mathrm{0,}\,{C}_{{\rm{spin}}}=-\,4.$$

The vanishing total Chern number *C* in the subsystems reflects the TR symmetry of Maxwell’s equations and the absence of QH states in free photons, while the spin Chern numbers *C*_spin_ = ±4 indicate that there exist two pairs of QSH edge states which are doubly-degenerate with respect to the helicity *σ*. The existence of surface modes in Maxwell’s equations, however, requires the presence of an interface (between two different media) that breaks the duality symmetry of electromagnetic fields as in an unbounded region, and therefore only one pair of edge modes survives at the interface^48^. This feature will be confirmed by the characteristic equation of surface modes at the interface between vacuum and the metamaterial.

According to Eqs (7) and (9), the eigensystem of the combined hybrid modes is written as

11 $$(\begin{array}{cc}{ {\mathcal H} }_{+} & 0\\ 0 & { {\mathcal H} }_{-}\end{array})(\begin{array}{c}{\psi }_{\sigma }\\ {\psi }_{-\sigma }\end{array})={\lambda }_{\sigma }(\begin{array}{c}{\psi }_{\sigma }\\ {\psi }_{-\sigma }\end{array}),$$

which states that the eigenstates *ψ*_*σ*_ and *ψ*_−*σ*_ of their respective subsystems are degenerate with the eigenvalue *λ*_*σ*_. The combined Hamiltonian is therefore considered two copies of the spin-orbit Hamiltonian with opposite helicity. This feature will play a crucial role in constructing the Kramers degeneracy in the present problem, leading to the topological protection of helical edge states.

### Pseudo time-reversal symmetry

The Hamiltonian for Maxwell’s equations [cf. Eq. (1)] in a lossless medium with $\underline{\varepsilon }={\underline{\varepsilon }}^{\ast }$ and $\underline{\mu }={\underline{\mu }}^{\ast }$ is time-reversal (TR) invariant under *T*_*b*_, that is,

12 $${T}_{b}{ {\mathcal H} }_{m}{T}_{b}^{-1}={ {\mathcal H} }_{m},\,{ {\mathcal H} }_{m}=(\begin{array}{cc}\omega \underline{\varepsilon } & c{\bf{k}}\times \underline{I}\\ c{\bf{k}}\times \underline{I} & -\omega \underline{\mu }\end{array}),$$

where *T*_*b*_ = *σ*_*z*_*K* with ${T}_{b}^{2}=\underline{I}$ is the bosonic TR operator for photons and *K* is the complex conjugation^18^. The Hamiltonian ${ {\mathcal H} }_{m}$, however, is not TR invariant under *T*_*f*_, that is, ${T}_{f}{ {\mathcal H} }_{m}{T}_{f}^{-1}\ne { {\mathcal H} }_{m}$, where *T*_*f*_ = *iσ*_*y*_*K* with ${T}_{f}^{2}=-\,\underline{I}$ is the fermionic TR operator for electrons^18^. Therefore, the Kramers degeneracy does not hold in a general photonic system, unless other symmetry such as polarization degeneracy^15,21^ or spatial symmetry^20^ has been imposed in the system.

In another aspect, the combined Hamiltonian of the hybrid modes for Maxwell’s equations with the degenerate condition: $\underline{\varepsilon }=-\,\underline{\mu }$ (at the reference frequency *ω*_0_) is TR invariant under *T*_*p*_, that is,

13 $${T}_{p}{ {\mathcal H} }_{c}{T}_{p}^{-1}={ {\mathcal H} }_{c},\,{ {\mathcal H} }_{c}=(\begin{array}{cc}{ {\mathcal H} }_{+} & 0\\ 0 & { {\mathcal H} }_{-}\end{array}),$$

where *T*_*p*_ is the fermionic-like *pseudo* TR operator having the same form of *T*_*f*_. The pseudo TR operator *T*_*p*_ is inspired by noticing that **E** + **H**′ ↔ **E** − **H**′ during the TR operation. The pseudo TR operator is thus defined as *T*_*p*_ = *T*_*b*_*σ*_*x*_ = *σ*_*z*_*Kσ*_*x*_ = *iσ*_*y*_*K* with ${T}_{p}^{2}=\,-\,\underline{I}$^21^. Here, *σ*_*x*_ = (0, 1; 1, 0), *σ*_*y*_ = (0, −*i*; *i*, 0), and *σ*_*z*_ = diag(1, −1) are the Pauli matrices. The pseudo TR symmetry of the combined Hamiltonian ${ {\mathcal H} }_{c}$ ensures the Kramers degeneracy and guarantees the appearance of a Kramers doublet.

As revealed in Eq. (11), the photonic Kramers doublet consists of an eigenstate *ψ*_*σ*_ of ${ {\mathcal H} }_{+}$ and another eigenstate *ψ*_−*σ*_ of ${ {\mathcal H} }_{-}$, with the same eigenvalue *λ*_*σ*_ (*σ* = ±1). The states *ψ*_*σ*_ and *ψ*_−*σ*_ become TR partners under *T*_*p*_, that is, ${T}_{p}{\psi }_{\sigma }={\psi }_{-\sigma }^{\ast }$ and ${T}_{p}{\psi }_{-\sigma }=-{\psi }_{\sigma }^{\ast }$. In addition, *ψ*_*σ*_ and *ψ*_−*σ*_ are orthogonal: $\langle {\psi }_{\sigma }|{\psi }_{-\sigma }\rangle =0$ [cf. Eq. (30) in Methods B], which implies that it is impossible to introduce any backscattering between the two states, unless the TR symmetry has been broken. The two states therefore counterpropagate toward opposite directions without backscattering, a typical feature of the helical edge states that appear in the QSH system. As indicated in the field basis *ψ* [cf. Eq. (4)], the photonic Kramers doublet is a pair of two pseudospin states for the hybrid modes, analogous to the spin-up and spin-down states in electronic systems. The nonzero Chern numbers *C*_*σ*_ associated with the eigenstate *ψ*_*σ*_ further assert that the helical edge states are topologically protected, their existence being guaranteed by the difference of topological invariants on two sides of the interface. As the topological invariants remain constant under arbitrary continuous deformations of the system, the topological properties of the isotropic medium will be preserved when a certian anisotropy is included in the medium. The exact calculation of topological invariants for the anisotropic medium may resort to the numerical integration of Berry curvatures^49^.

### Surface modes

Let the *xz* plane be the interface between a dielectric with the relative material parameters *ε*_*d*_, *μ*_*d*_ and a uniaxially anisotropic medium characterized by *ε*_*t*_, *ε*_*z*_, *μ*_*t*_, and *μ*_*z*_. The characteristic equation of surface modes is formulated based on Maxwell’s boundary conditions: the continuity of tangential electric and magnetic field components at the interface, which is given by (see Methods C)

14 $$\begin{array}{c}({\varepsilon }_{t}{\mu }_{t}-{\varepsilon }_{d}{\mu }_{d}){k}_{z}^{2}({k}_{x}^{2}+{k}_{z}^{2}-{\varepsilon }_{t}{\mu }_{t}{k}_{0}^{2})-{\varepsilon }_{t}{\mu }_{t}{k}_{0}^{2}({k}_{y}^{(1)}{\varepsilon }_{t}-{k}_{y}^{(4)}{\varepsilon }_{d})({k}_{y}^{(2)}{\mu }_{t}-{k}_{y}^{(3)}{\mu }_{d})\\ \,\,\,\,\,\,+{k}_{z}^{2}[{\varepsilon }_{t}{\mu }_{t}({k}_{y}^{(1)}{k}_{y}^{(2)}+{k}_{y}^{(3)}{k}_{y}^{(4)})-({\varepsilon }_{t}{\mu }_{d}{k}_{y}^{(1)}{k}_{y}^{(3)}+{\varepsilon }_{d}{\mu }_{t}{k}_{y}^{(2)}{k}_{y}^{(4)})]=0,\end{array}$$

where ${k}_{y}^{\mathrm{(1)}}={k}_{y}^{\mathrm{(2)}}=\sqrt{{\varepsilon }_{d}{\mu }_{d}{k}_{0}^{2}-{k}_{x}^{2}-{k}_{z}^{2}}$ are the normal (to interface) wave vector components in the dielectric, and ${k}_{y}^{\mathrm{(3)}}=-\,\sqrt{{\varepsilon }_{z}{\mu }_{t}{k}_{0}^{2}-{k}_{x}^{2}-\frac{{\varepsilon }_{z}}{{\varepsilon }_{t}}{k}_{z}^{2}}$ and ${k}_{y}^{\mathrm{(4)}}=-\,\sqrt{{\varepsilon }_{t}{\mu }_{z}{k}_{0}^{2}-{k}_{x}^{2}-\frac{{\mu }_{z}}{{\mu }_{t}}{k}_{z}^{2}}$ are the normal components in the anisotropic medium. For the surface waves to be valid on the dielectric side (*y* > 0), ${k}_{y}^{\mathrm{(1)}}$ and ${k}_{y}^{\mathrm{(2)}}$ should be purely imaginary with a positive value, which requires that ${k}_{x}^{2}+{k}_{z}^{2} > {\varepsilon }_{d}{\mu }_{d}{k}_{0}^{2}$. On the anisotropic medium side (*y* < 0), ${k}_{y}^{\mathrm{(3)}}$ and ${k}_{y}^{\mathrm{(4)}}$ should be purely imaginary with a negative value, which requires that ${k}_{x}^{2}+\frac{{\varepsilon }_{z}}{{\varepsilon }_{t}}{k}_{z}^{2} > {\varepsilon }_{z}{\mu }_{t}{k}_{0}^{2}$ and ${k}_{x}^{2}+\frac{{\mu }_{z}}{{\mu }_{t}}{k}_{z}^{2} > {\varepsilon }_{t}{\mu }_{z}{k}_{0}^{2}$. In the presence of square roots in ${k}_{y}^{(n)}$ (*n* = 1, 2, 3, 4), the surface modes are represented by a part of the bi-quadratic surface in the frequency-wave vector space. In the isotropic case, where *ε*_*t*_ = *ε*_*z*_ ≡ *ε* and *μ*_*t*_ = *μ*_*z*_ ≡ *μ*, Eq. (14) is simplified to

15 $${k}_{x}^{2}+{k}_{z}^{2}=\frac{\varepsilon {\varepsilon }_{d}(\varepsilon {\mu }_{d}-\mu {\varepsilon }_{d})}{{\varepsilon }^{2}-{\varepsilon }_{d}^{2}}{k}_{0}^{2}\,{\rm{or}}\,{k}_{x}^{2}+{k}_{z}^{2}=\frac{\mu {\mu }_{d}(\mu {\varepsilon }_{d}-\varepsilon {\mu }_{d})}{{\mu }^{2}-{\mu }_{d}^{2}}{k}_{0}^{2},$$

depending on whether $\varepsilon ({\omega }_{0}) < 0$ or $\mu ({\omega }_{0}) < 0$, respectively. In this situation, the characteristic equation represents a quadratic surface of revolution about the frequency axis.

At the reference frequency *ω*_0_, where *ε*_*t*_ = −*μ*_*t*_ and *ε*_*z*_ = −*μ*_*z*_, Eq. (14) is reduced to

16 $$\begin{array}{c}({\mu }_{t}^{2}-{\varepsilon }_{d}{\mu }_{d}){k}_{x}^{2}+({\mu }_{t}{\mu }_{z}-{\varepsilon }_{d}{\mu }_{d}){k}_{z}^{2}-{\varepsilon }_{d}{\mu }_{d}{\mu }_{t}({\mu }_{t}+{\mu }_{z}){k}_{0}^{2}\\ +{\mu }_{t}({\varepsilon }_{d}-{\mu }_{d})\sqrt{{k}_{x}^{2}+{k}_{z}^{2}-{\varepsilon }_{d}{\mu }_{d}{k}_{0}^{2}}\sqrt{{k}_{x}^{2}+\frac{{\mu }_{z}}{{\mu }_{t}}{k}_{z}^{2}+{\mu }_{z}{\mu }_{t}{k}_{0}^{2}}=0,\end{array}$$

which is a part of the bi-quadratic curve. If *ε*_*d*_ = *μ*_*d*_, Eq. (16) is further simplified to a standard quadratic curve as

17 $$({\mu }_{t}^{2}-{\mu }_{d}^{2}){k}_{x}^{2}+({\mu }_{t}{\mu }_{z}-{\mu }_{d}^{2}){k}_{z}^{2}={\mu }_{d}^{2}{\mu }_{t}({\mu }_{t}+{\mu }_{z}){k}_{0}^{2},$$

which represents an ellipse when ${\mu }_{t}^{2} > {\mu }_{d}^{2}$ and ${\mu }_{t}{\mu }_{z} > {\mu }_{d}^{2}$ or a two-sheeted hyperbola when ${\mu }_{t}{\mu }_{z} < {\mu }_{d}^{2} < {\mu }_{t}^{2}$ or ${\mu }_{t}^{2} < {\mu }_{d}^{2} < {\mu }_{t}{\mu }_{z}$. Here, we assume that *μ*_*t*_ and *μ*_*z*_ are of the same sign. In the isotropic case, Eq. (15) at the reference frequency *ω*_0_ is simplified to

18 $${k}_{x}^{2}+{k}_{z}^{2}=\frac{{\varepsilon }^{2}{\varepsilon }_{d}({\varepsilon }_{d}+{\mu }_{d})}{{\varepsilon }^{2}-{\varepsilon }_{d}^{2}}{k}_{0}^{2}\,{\rm{or}}\,{k}_{x}^{2}+{k}_{z}^{2}=\frac{{\mu }^{2}{\mu }_{d}({\varepsilon }_{d}+{\mu }_{d})}{{\mu }^{2}-{\mu }_{d}^{2}}{k}_{0}^{2},$$

depending on whether $\varepsilon  < 0$ or $\mu  < 0$, respectively. In this situation, the characteristic equation represents a circle when ${\varepsilon }^{2} > {\varepsilon }_{d}^{2}$ or ${\mu }^{2} > {\varepsilon }_{d}^{2}$, which is valid even when *ε*_*d*_ ≠ *μ*_*d*_. In case all the materials considered are nonmagnetic, that is, *μ* = 1 (*ε* = −1) and *μ*_*d*_ = 1 at *ω* = *ω*_0_, Eq. (16) is simplified to ${k}_{x}^{2}+{k}_{z}^{2}={\varepsilon }_{d}{k}_{0}^{2}/(1-{\varepsilon }_{d})$, which represents a circle when $0 < {\varepsilon }_{d} < 1$.

For a fixed *k*_*z*_ (*k*_*x*_), Eq. (16) allows for two solutions of *k*_*x*_ (*k*_*z*_) with reflection symmetry about the *k*_*z*_ (*k*_*x*_) axis, which means that there exist a pair of surface modes at the interface counterpropagating toward the positive and negative *k*_*x*_ (*k*_*z*_) directions.

## Discussion

**Figure 1:**
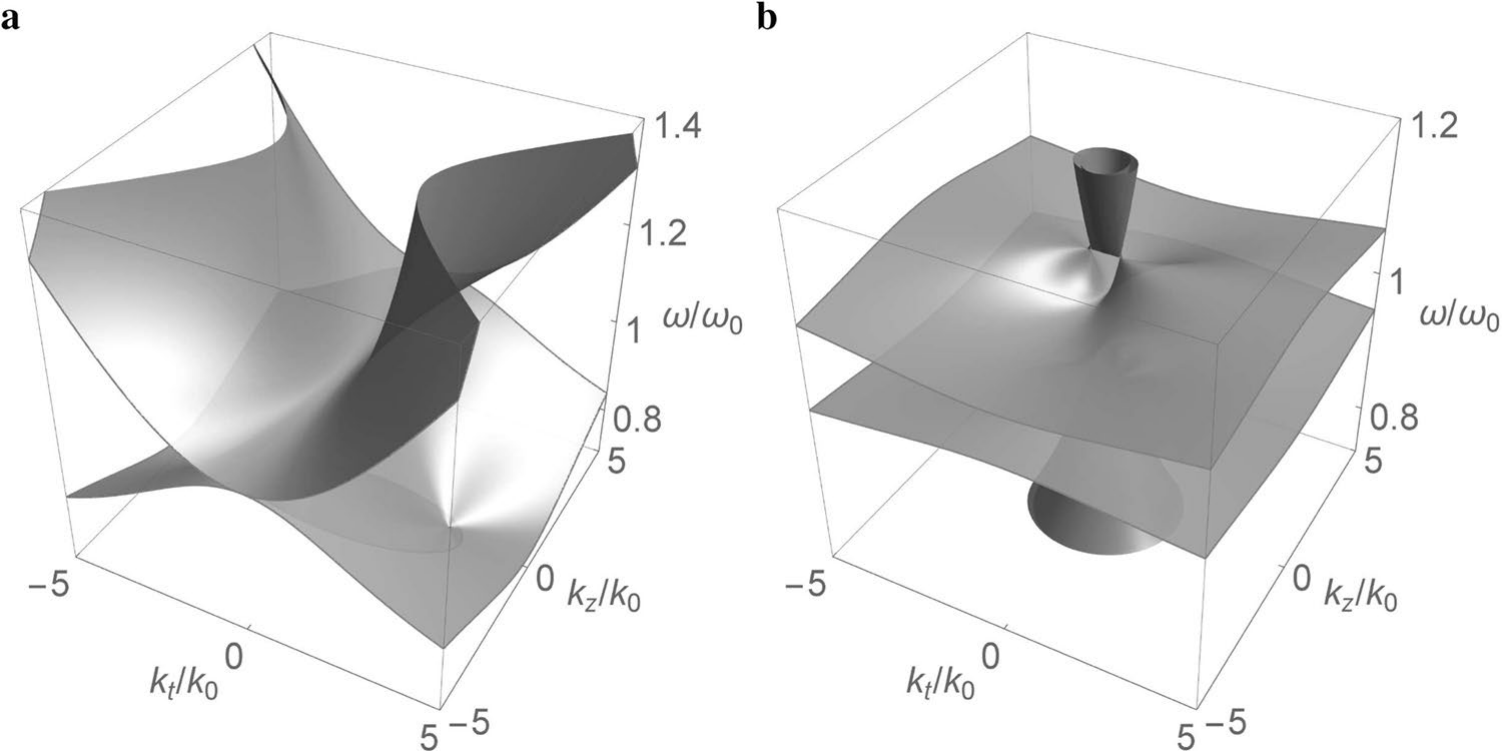
Dispersion of bulk modes in the frequency-wave vector space for the dispersive metamaterial based on Eq. (2) with *ω*_*p*_/*ω*_0_ = 1.5 and (**a**) *ε*_*t*∞_ = 0.25, *μ*_*t*∞_ = 4.25, *ε*_*z*∞_ = *μ*_*z*∞_ = 2.25 (*ε*_*t*_ = −*μ*_*t*_ = −2, *ε*_*z*_ = *μ*_*z*_ = 0 at *ω* = *ω*_0_) (**b**) *ε*_*t*∞_ = 1.9, *ε*_*z*∞_ = 2.1, *μ*_*t*∞_ = 2.6, *μ*_*z*∞_ = 2.4 (*ε*_*t*_ = −*μ*_*t*_ = −0.35, *ε*_*z*_ = −*μ*_*z*_ = −0.15 at *ω* = *ω*_0_).

Figure 1 shows the dispersion of bulk modes in the frequency-wave vector space for the dispersive medium based on Eq. (2). The Drude-type dispersive model, which is usually employed in the analysis of metamaterials, is assumed for the permittivity and permeability components: ${\varepsilon }_{n}={\varepsilon }_{n\infty }-{\omega }_{ep}^{2}/{\omega }^{2}$ and ${\mu }_{n}={\mu }_{n\infty }-{\omega }_{mp}^{2}/{\omega }^{2}$ (*n* = *t*, *z*)^50,51^, where *ω*_*ep*_ and *ω*_*mp*_ are the effective plasma frequency and magnetic plasma frequency, respectively^52,53^. For simplicity, we further assume that *ω*_*ep*_ = *ω*_*mp*_≡*ω*_*p*_^50,51^. If *ε*_*z*_ = 0 or *ε*_*t*_ = 0 at the reference frequency *ω*_0_, the upper and the lower modes touch at *ω* = *ω*_0_ [Fig. 1(a)]. In particular, the upper mode has a positive index (along the optical axis): ${n}_{z}\equiv \sqrt{{\varepsilon }_{z}{\mu }_{z}} > 0$, while the lower mode has a negative index: ${n}_{z} < 0$.

If both *ε*_*t*_ ≠ 0 and *ε*_*z*_ ≠ 0 at *ω* = *ω*_0_, a frequency gap is opened between the two bulk modes, with the gap size dependent on the material parameters [Fig. 1(b)]. As the anisotropy of the medium is reduced, the bulk modes tend to be dispersionless, that is, independent of the frequency. In the isotropic case, where *ε*_*t*∞_ = *ε*_*z*∞_ ≡ *ε*_∞_ and *μ*_*t*∞_ = *μ*_*z*∞_ ≡ *μ*_∞_, the bulk modes are basically represented by two flat surfaces at $\omega ={\omega }_{p}/\sqrt{{\varepsilon }_{\infty }}$ and $\omega ={\omega }_{p}/\sqrt{{\mu }_{\infty }}$, leaving in between a frequency gap when *ε*_∞_ ≠ *μ*_∞_.

**Figure 2:**
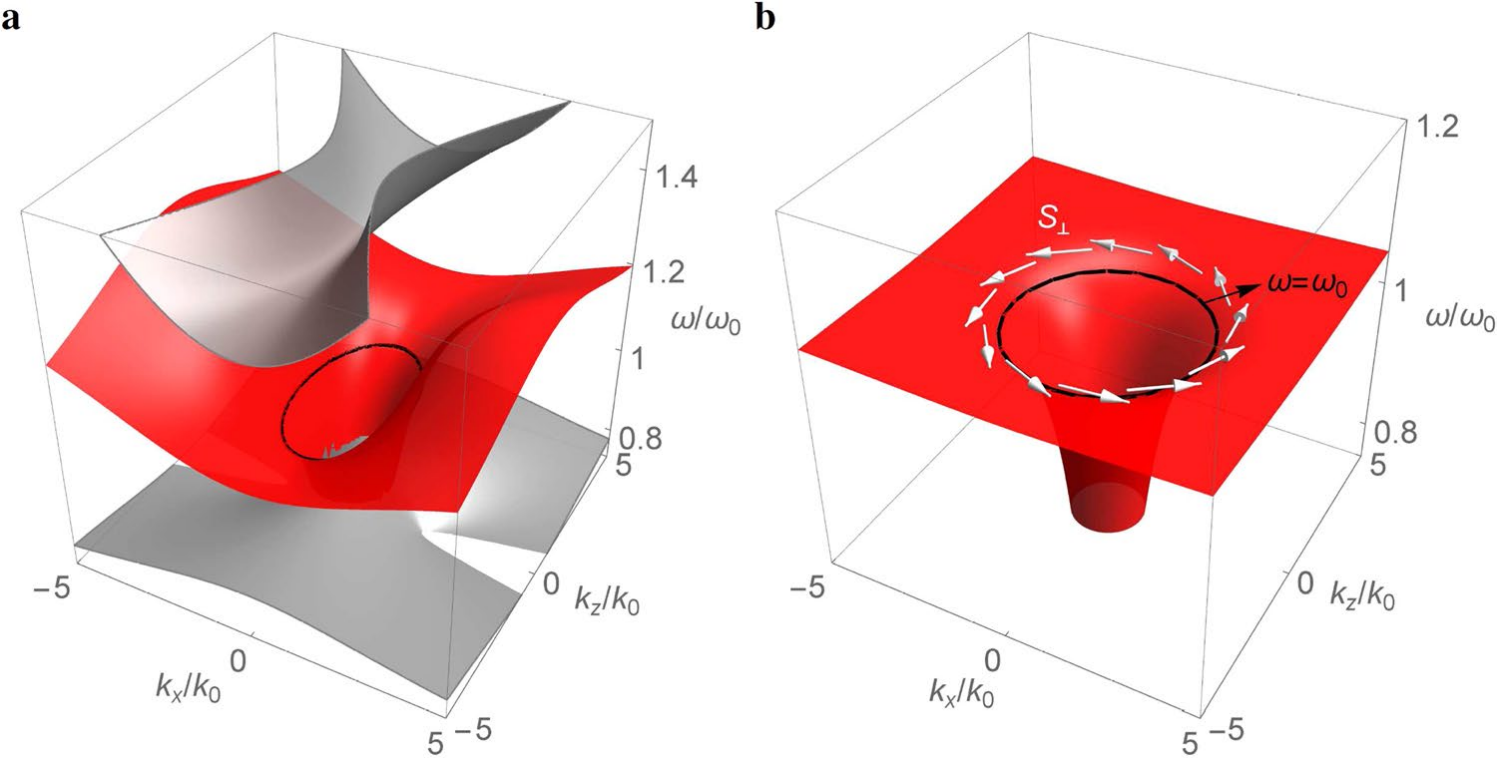
Dispersion of surface modes (in red color) at the interface between vacuum and the dispersive metamaterial based on Eq. (14) with *ω*_*p*_/*ω*_0_ = 1.5 and (**a**) *ε*_*t*∞_ = 0.25, *ε*_*z*∞_ = 1.25, *μ*_*t*∞_ = 4.25, *μ*_*z*∞_ = 3.25 (*ε*_*t*_ = −*μ*_*t*_ = −2 and *ε*_*z*_ = −*μ*_*z*_ = −1 at *ω* = *ω*_0_) and (**b**) *ε*_*t*∞_ = *ε*_*z*∞_ = 1.05 and *μ*_*t*∞_ = *μ*_*z*∞_ = 3.45 (*ε*_*t*_ = *ε*_*z*_ = −*μ*_*t*_ = −*μ*_*z*_ = −1.2 at *ω* = *ω*_0_). Black curves are surface modes at *ω* = *ω*_0_. Gray surfaces in (**a**) are bulk modes. White arrows in (**b**) denote the transverse spin **S**_⊥_.

Figure 2(a) shows the dispersion of surface mode (in red color) at the interface between vacuum and the dispersive metamaterial based on Eq. (14), which exists in the frequency gap between two bulk modes (in gray color). The surface mode is represented by a funnel-shaped surface with a lower frequency at the center that connects to the lower bulk mode. As *k*_*x*_ or *k*_*z*_ increases, the surface mode raises its frequency and approaches asymptotically to

19 $${\omega }_{sx}=\frac{{\omega }_{p}}{\sqrt{{\varepsilon }_{d}+{\varepsilon }_{t\infty }}}\,{\rm{or}}\,{\omega }_{sz}={\omega }_{p}\sqrt{\frac{(\sqrt{{({\varepsilon }_{t\infty }-{\varepsilon }_{z\infty })}^{2}+4{\varepsilon }_{d}^{2}}-{\varepsilon }_{t\infty }-{\varepsilon }_{z\infty })}{2({\varepsilon }_{d}^{2}-{\varepsilon }_{t\infty }{\varepsilon }_{z\infty })}},$$

respectively, which are obtained by taking the limit as *k*_*x*_ → ∞ or *k*_*z*_ → ∞ in the characteristic equation of surface mode [cf. Eq. (14)]. There may exist a gap between the surface mode and the upper bulk mode, depending on the constitutive parameters of the metamaterial. For ${\varepsilon }_{z\infty } > {\varepsilon }_{t\infty }$ (${\varepsilon }_{z\infty } < {\varepsilon }_{t\infty }$), the lowest frequency of the upper bulk mode is given by ${\omega }_{b}={\omega }_{p}/\sqrt{{\varepsilon }_{z\infty }}$ (${\omega }_{b}={\omega }_{p}/\sqrt{{\varepsilon }_{t\infty }}$). A gap is opened when ${\omega }_{b} > {\omega }_{sx}$ (${\omega }_{b} > {\omega }_{sz}$), where we also have ${\omega }_{sz} < {\omega }_{sx}$ (${\omega }_{sx} < {\omega }_{sz}$). At the reference frequency *ω*_0_, where the degenerate condition *ε* = −*μ* is satisfied, the surface mode is described by an ellipse (denoted by black curve) with *ε*_*d*_ = *μ*_*d*_ and ${\mu }_{t}^{2} > {\mu }_{t}{\mu }_{z} > {\mu }_{d}^{2}$ [cf. Eq. (17)]. In the isotropic case where *ε*_*t*∞_ = *ε*_*z*∞_ = *ε*_∞_, the surface mode approaches asymptotically to a flat surface for a sufficiently large *k*_*x*_ or *k*_*z*_, as shown in Fig. 2(b). The asymptotic frequency becomes ${\omega }_{s}={\omega }_{p}/\sqrt{{\varepsilon }_{d}+{\varepsilon }_{\infty }}$ [cf. Eq. (19)], which has the same form of the surface plasma frequency at the interface between a dielectric and the Drude-type metal. In this situation, there is always a gap between the surface mode and the upper bulk mode. At the reference frequency *ω*_0_, the surface mode is represented by a circle [cf. Eq. (18)].

**Figure 3:**
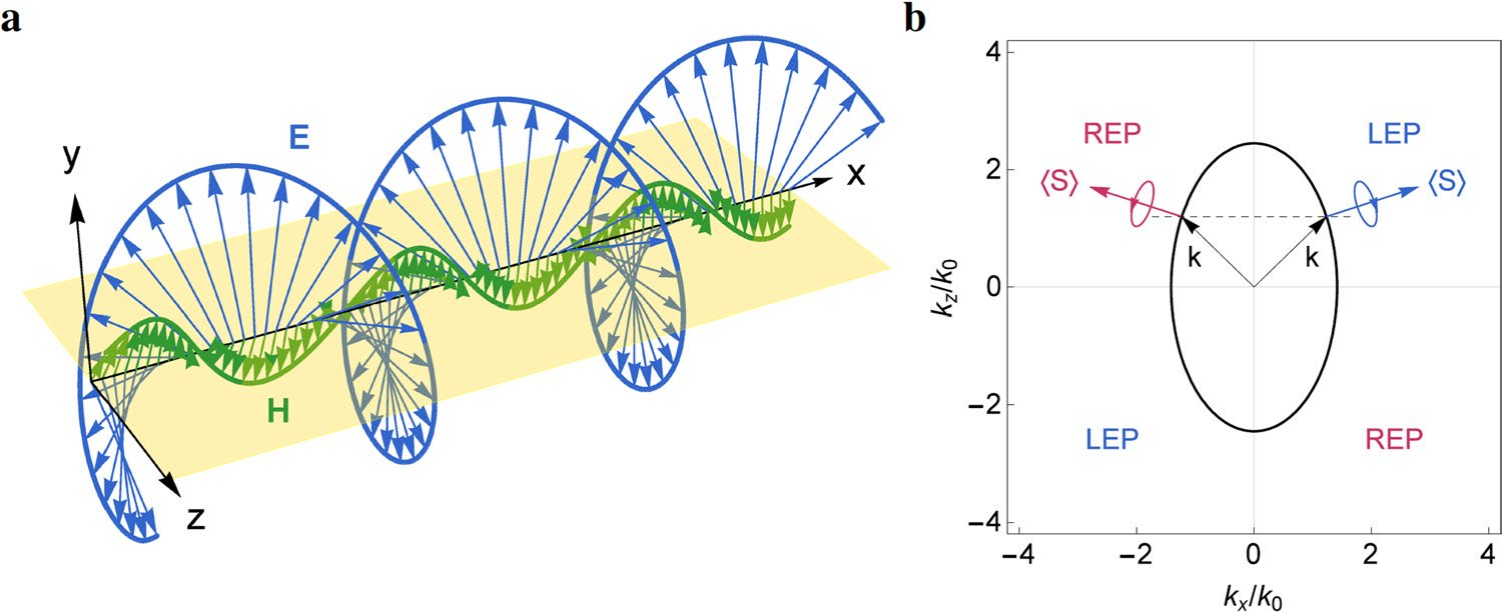
(**a**) Instantaneous electric field ${\bf{E}}({\bf{r}},t)={\rm{Re}}[{\bf{E}}({\bf{r}}){e}^{-i\omega t}]$ and magnetic field ${\bf{H}}({\bf{r}},t)={\rm{Re}}[{\bf{H}}({\bf{r}}){e}^{-i\omega t}]$ for the surface wave at the interface between vacuum ($y > 0$) and the dispersive metamaterial ($y < 0$) at the reference frequency *ω*_0_ with the same parameters in Fig. 2(a). Light yellow plane denotes the interface. (**b**) Polarization handedness of the same surface wave for different signs of *k*_*x*_ and *k*_*z*_. Black dashed line indicates the constant *k*_*z*_ = 1.2*k*_0_. Black arrows are wave vectors. Blue and red arrows denote the energy flows for ${k}_{x} > 0$ and ${k}_{x} < 0$, respectively.

Since the surface waves are evanescent in the direction normal to the interface, their normal wave vector components are purely imaginary, that is, *k*_*y*_ = ±*iκ*_*y*_ and *κ*_*y*_ is real. The transversality condition (*k* ⋅ *E* = 0) dictates that the normal (to interface) electric field component *E*_*y*_ has a *π*/2 phase difference relative to the tangential component *E*_*x*_ or *E*_*z*_. As the wave propagates at the interface (*y* = 0), the electric fields rotate in the perpendicular (*xy* or *yz*) plane, leading to elliptically polarized waves. The elliptical polarization of the surface waves is demonstrated in Fig. 3(a), where the instantaneous electric fields (blue arrows) and magnetic fields (green arrows) at the interface result in helical trajectories (traced by the tips of field vectors) along the propagation direction (the helical trajectory for the magnetic fields is less obvious as the normal magnetic field componet *H*_*y*_ is relatively smaller than the electric field component *E*_*y*_). In particular, the rotating electric field generates a *transverse spin* **S**_⊥_ perpendicular to the wave vector **k** as^54^

20 $${{\bf{S}}}_{\perp }=\frac{{\rm{Re}}[{\bf{k}}]\times {\rm{Im}}[{\bf{k}}]}{{({\rm{Re}}[{\bf{k}}])}^{2}},$$

which is considered as the spin-momentum locking in the evanescent surface waves. The transverse spin of the surface mode exhibits a vortex spin texture [denoted by white arrows in Fig. 2(b)] that occurs in the surface states for 3D topological insulators.

For the evanescent surface wave with the transverse spin, the handedness can be evaluated by first calculating the electric field components in a new coordinate system (*x*′, *y*′, *z*′), obtained by rotating the original system (*x*, *y*, *z*) about the *y* axis such that the *x*′ axis is oriented to the time-averaged Poynting vector on the *xz* plane. Denoting *θ* the angle from the *x* axis to the *x*′ axis, the electric field components in the new coordinate system are given by *E*_*x*′_ = *E*_*x*_cos*θ* + *E*_*z*_sin*θ*, *E*_*y*′_ = *E*_*y*_, and *E*_*z*′_ = −*E*_*x*_sin*θ* + *E*_*z*_cos*θ*. The polarization handedness is then determined by the phase difference *δ* between *E*_*y*′_ and *E*_*z*′_: *δ* = *arg*(*E*_*z*′_) − *arg*(*E*_*y*′_), where *arg*(*z*) ≡ *arctan*(I*m*[*z*]/R*e*[*z*]) is the argument of a complex number *z*. The wave is right-handed elliptically polarized (REP) or left-handed elliptically polarized (LEP) if *δ* = *π*/2 or −*π*/2, respectively, that is, the phase of *E*_*z*′_ is delayed or advanced by 90° relative to that of *E*_*y*′_ (under the time-harmonic convention *e*^−*iωt*^).

In Fig. 3(b), the polarization handedness of the surface waves at the reference frequency *ω*_0_ is shown to have odd symmetry with respect to either of the two in-plane wave vector components (*k*_*x*_ and *k*_*z*_). For a constant *k*_*z*_ (indicated by black dashed line), there exist a LEP wave propagating toward the positive *k*_*x*_ direction and a REP wave toward the negative *k*_*x*_ direction. The two surface waves with opposite handedness counterpropagate at a given edge (*y* = 0 in the *xy* plane), showing the feature of spin-momentum locking that occurs in the helical edge states. This feature holds when the surface mode is described by either an ellipse (for the anisotropic medium) or a circle (for the isotropic medium).

**Figure 4:**
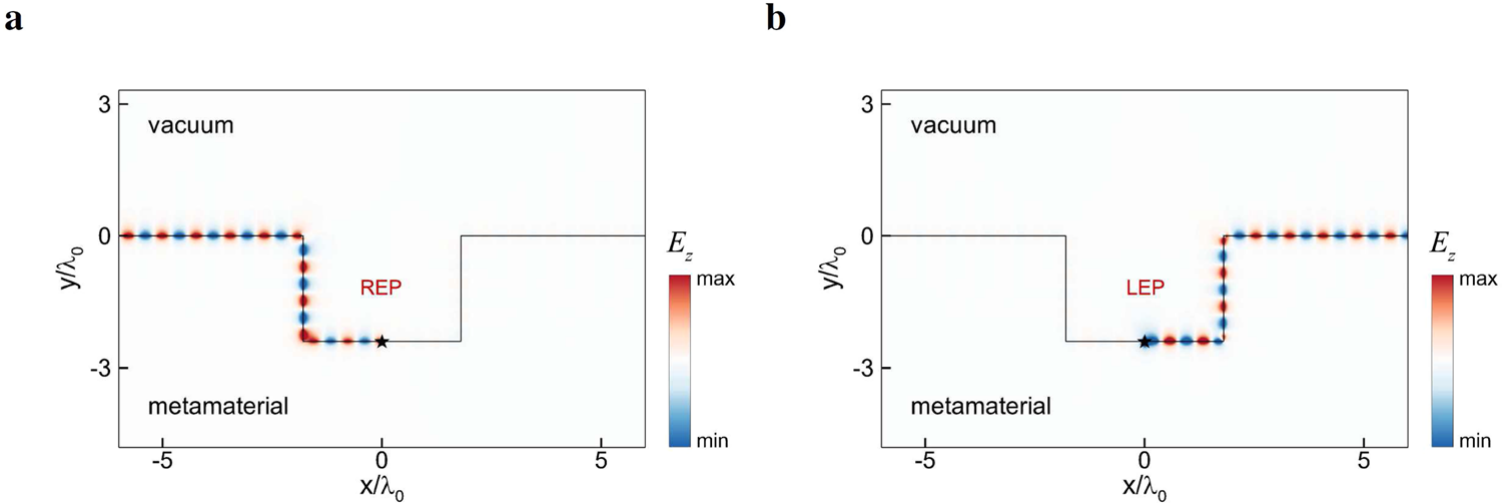
Electromagnetic wave simulation for the surface modes at the interface between vacuum and the dispersive metamaterial with the same paramsters in Fig. 3(b). Asteristic symbols denote the dipole sources for exciting the (**a**) REP and (**b**) LEP surface waves at the reference frequency *ω*_0_.

Finally, the topological properties of the surface modes are illustrated with the electromagnetic wave propagation at the interface between vacuum and the metamaterial^55^, as shown in Fig. 4. Here, a dipole source with *k*_*z*_/*k*_0_ = 1.2 is placed at the interface (marked by asterisk symbol) to excite the surface wave, where the field is evanescent both in vacuum (outside the dispersion circle: ${k}_{x}^{2}+{k}_{z}^{2} > {k}_{0}^{2}$) and the metamaterial (inside the frequency gap). By appropriately adjusting the phase of the point dipole, the REP (LEP) surface wave at the reference frequency *ω*_0_ propagates unidirectionally toward the left (right), which is consistent with the direction of surface wave energy flow [cf. Figure 3(b)] and exhibits the typical feature of spin-polarized helical edge states. In particular, the surface waves are able to bend around sharp corners without backscattering, showing the robust transport of edge states against disorder. As the frequency deviates from *ω*_0_, the degenerate condition (*ε* = −*μ*) will be violated to a certain extent. The removal of degeneracy, however, is tolerated to some degree for the system to remain in the photonic topological phase^15,23^.

In conclusion, we have analyzed the photonic topological phases in dispersive metamaterials with the degenerate condition at a reference frequency. The topological phases are characterized by the nonzero spin Chern numbers for the hybrid modes described by the spin-orbit Hamiltonians with pseudospin 1. In particular, the hybrid modes comply with a fermionic-like pseudo TR symmetry that ensures the Kramers degeneracy, leading to the topological protection of helical edge states. The transverse spin generated by the evanescent surface wave is perpendicular to the wave vector, which exhibits the spin-momentum locking as in the surface states for 3D topological insulators. The topological features of helical edge states are further demonstrated by the robust transport of surface waves at an irregular boundary between vacuum and the dispersive metamaterial.

## Methods

### Spin-orbit Hamiltonians

The wave equation for the plus hybrid mode **F** = **E** + **H**′ in Eq. (3) can be rewritten as

21 $${ {\mathcal H} }_{+}\tilde{\psi }=\tilde{D}\tilde{\psi },$$

where

22 $${ {\mathcal H} }_{+}=c(\begin{array}{lll}{\kappa }_{z} & \frac{{\kappa }_{x}-i{\kappa }_{y}}{\sqrt{2}} & 0\\ \frac{{\kappa }_{x}+i{\kappa }_{y}}{\sqrt{2}} & 0 & \frac{{\kappa }_{x}-i{\kappa }_{y}}{\sqrt{2}}\\ 0 & \frac{{\kappa }_{x}+i{\kappa }_{y}}{\sqrt{2}} & -{\kappa }_{z}\end{array}),\,\tilde{D}=\omega (\begin{array}{lll}{\varepsilon }_{t} & 0 & 0\\ 0 & {\varepsilon }_{z} & 0\\ 0 & 0 & {\varepsilon }_{t}\end{array}),$$

and $\tilde{\psi }={(\frac{-{F}_{x}+i{F}_{y}}{\sqrt{2}},{F}_{z},\frac{{F}_{x}+i{F}_{y}}{\sqrt{2}})}^{T}$ is the basis of the *pseudospin* states that include a *π*/2 phase difference in the transverse field components (with respect to the optical axis of the anisotropic medium)^45^. In the neighborhood of *ω*_0_, *ε*_*n*_ is approximated as $\omega {\varepsilon }_{n}\approx {\omega }_{0}{\varepsilon }_{n0}+{\tilde{\varepsilon }}_{n}\delta \omega$ (*n* = *t*, *z*) and Eq. (21) can be rearranged as

23 $${ {\mathcal H} }_{+}\psi -D\psi =\delta \omega \psi ,$$

where

24 $${ {\mathcal H} }_{+}=\frac{c}{\sqrt{{\tilde{\varepsilon }}_{t}{\tilde{\varepsilon }}_{z}}}(\begin{array}{lll}\sqrt{\frac{{\tilde{\varepsilon }}_{z}}{{\tilde{\varepsilon }}_{t}}}{\kappa }_{z} & \frac{{\kappa }_{x}-i{\kappa }_{y}}{\sqrt{2}} & 0\\ \frac{{\kappa }_{x}+i{\kappa }_{y}}{\sqrt{2}} & 0 & \frac{{\kappa }_{x}-i{\kappa }_{y}}{\sqrt{2}}\\ 0 & \frac{{\kappa }_{x}+i{\kappa }_{y}}{\sqrt{2}} & -\sqrt{\frac{{\tilde{\varepsilon }}_{z}}{{\tilde{\varepsilon }}_{t}}}{\kappa }_{z}\end{array}),\,D={\omega }_{0}(\begin{array}{lll}\frac{{\varepsilon }_{t0}}{{\tilde{\varepsilon }}_{t}} & 0 & 0\\ 0 & \frac{{\varepsilon }_{t0}}{{\tilde{\varepsilon }}_{z}} & 0\\ 0 & 0 & \frac{{\varepsilon }_{t0}}{{\tilde{\varepsilon }}_{t}}\end{array}),$$

and $\psi ={U}^{-1}\tilde{\psi }$ with $U={\rm{diag}}(\sqrt{{\tilde{\varepsilon }}_{z}/{\tilde{\varepsilon }}_{t}},1,\sqrt{{\tilde{\varepsilon }}_{z}/{\tilde{\varepsilon }}_{t}})$. In the isotropic case, where *ε*_*t*0_ = *ε*_*z*0_ ≡ *ε* and ${\tilde{\varepsilon }}_{t}={\tilde{\varepsilon }}_{z}\equiv \tilde{\varepsilon }$, Eq. (23) is simplified to

25 $${ {\mathcal H} }_{+}\psi -d\psi =\delta \omega \psi ,$$

where $$d={\omega }_{0}\varepsilon /\tilde{\varepsilon }$$ and

26 $${ {\mathcal H} }_{+}=\alpha S\cdot \kappa ,$$

with $$\alpha =c/\tilde{\varepsilon }$$, $$\kappa ={\kappa }_{x}\hat{x}+{\kappa }_{y}\hat{y}+{\kappa }_{z}\hat{z}$$, $${\bf{S}}={S}_{x}\hat{x}+{S}_{y}\hat{y}+{S}_{z}\hat{z}$$, and

27 $${S}_{x}=\frac{1}{\sqrt{2}}(\begin{array}{lll}0 & 1 & 0\\ 1 & 0 & 1\\ 0 & 1 & 0\end{array}),\,{S}_{y}=\frac{1}{\sqrt{2}}(\begin{array}{lll}0 & -i & 0\\ i & 0 & -i\\ 0 & i & 0\end{array}),\,{S}_{z}=(\begin{array}{lll}1 & 0 & 0\\ 0 & 0 & 0\\ 0 & 0 & -1\end{array})$$

being the spin matrices for spin 1.

### Topological invariants

In terms of spherical coordinates, the Hamiltonian for the plus hybrid mode ${ {\mathcal H} }_{+}=\alpha S\cdot \kappa$ [cf. Eq. (26)] can be rewritten as

28 $${ {\mathcal H} }_{+}=\frac{|d|}{\sqrt{2}}(\begin{array}{lll}\sqrt{2}\,\cos \,\theta  & \sin \,\theta {e}^{-i\varphi } & 0\\ \sin \,\theta {e}^{i\varphi } & 0 & \sin \,\theta {e}^{-i\varphi }\\ 0 & \sin \,\theta {e}^{i\varphi } & -\sqrt{2}\,\cos \,\theta \end{array}),$$

where *κ*_*x*_ = *a *sin *θ *cos *ϕ*, *κ*_*y*_ = *a *sin *θ *sin *ϕ*, and *κ*_*z*_ = *a *cos *θ* with $a=|\varepsilon {k}_{0}|$. Here, *θ* and *ϕ* are the polar angle and azimuthal angle, respectively, on the closed surface *S*: ${\kappa }_{x}^{2}+{\kappa }_{y}^{2}+{\kappa }_{z}^{2}={\varepsilon }^{2}{k}_{0}^{2}$, corresponding to the bulk mode at the reference frequency *ω*_0_ in the imaginary wave vector space. The eigensystem of the plus hybrid mode

29 $${ {\mathcal H} }_{+}{\psi }_{\sigma }={\lambda }_{\sigma }{\psi }_{\sigma },$$

is solved to give the eigenvalues ${\lambda }_{\sigma }=|d|\sigma$ (*σ* = ±1, 0) and the normalized eigenvectors as

30 $${\psi }_{\sigma }=\frac{1}{2}(\begin{array}{c}\sigma {e}^{-2i\varphi }(\sigma +\,\cos \,\theta )\\ \sigma \sqrt{2}{e}^{-i\varphi }\,\sin \,\theta \\ 1-\sigma \,\cos \,\theta \end{array})\,(\sigma =\pm \,\mathrm{1),}$$

31 $${\psi }_{\sigma }=\frac{1}{\sqrt{2}}(\begin{array}{c}-{e}^{-2i\varphi }\,\sin \,\theta \\ \sqrt{2}{e}^{-i\varphi }\,\cos \,\theta \\ \sin \,\theta \end{array})\,(\sigma =\mathrm{0).}$$

Note here that the eigenvalue *λ*_*σ*_ is related to *δω* in Eq. (4) as *λ*_*σ*_ = *d* + *δω*, while the eigenfuntion *ψ*_*σ*_ is the same as *ψ*. Based on Eqs (30) and (31), the Berry connections ${{\bf{A}}}_{\sigma }=-\,i\langle {\psi }_{\sigma }|\nabla {\psi }_{\sigma }\rangle$ are obtained as

32 $${{\bf{A}}}_{\sigma }=-\,\frac{1}{r}\,\cot \,\frac{\theta }{2}\hat{\varphi }\,(\sigma =\,1),$$

33 $${{\bf{A}}}_{\sigma }=-\,\frac{1}{r}\,\tan \,\frac{\theta }{2}\hat{\varphi }\,(\sigma =-\,1),$$

34 $${{\bf{A}}}_{\sigma }=-\,\frac{1}{r}\csc \,\theta \hat{\varphi }\,(\sigma =0).$$

The Berry curvatures ${{\bf{F}}}_{\sigma }=\nabla \times {{\bf{A}}}_{\sigma }$ are thus given by

35 $${{\bf{F}}}_{\sigma }=\sigma \frac{\hat{r}}{{r}^{2}}\,(\sigma =\pm \,1,0).$$

Integrating over the closed sphere *S*, the Chern numbers ${C}_{\sigma }=\frac{1}{2\pi }{\int }_{S}{{\bf{F}}}_{\sigma }\cdot d{\bf{s}}$ are calculated to give

36 $${C}_{\sigma }=2\sigma \,(\sigma =\pm \,1,0).$$

### Surface wave equation

According to Maxwell’s equations, the eigenfields on either side of the interface (*y* = 0) are given by the nontrivial solutions of *E* and *H* [cf. Eq. (1)] or the *null space* of *H*_*m*_ [cf. Eq. (12)]. On the dielectric side ($y > 0$), we have

37 $${{\bf{H}}}^{\mathrm{(1)}}=\frac{1}{{k}_{0}{\eta }_{0}}({k}_{z},\,\mathrm{0,}-\,{k}_{x}),\,{{\bf{E}}}^{\mathrm{(1)}}=\frac{1}{{k}_{0}^{2}{\varepsilon }_{d}}({k}_{x}{k}_{y}^{\mathrm{(1)}},-\,{k}_{x}^{2}-{k}_{z}^{2},{k}_{y}^{\mathrm{(1)}}{k}_{z}),$$

38 $${{\bf{H}}}^{(2)}=\frac{1}{{k}_{0}{\eta }_{0}}({k}_{y}^{(2)},-\,{k}_{x},\,0),\,{{\bf{E}}}^{(2)}=-\,\frac{1}{{k}_{0}^{2}{\varepsilon }_{d}}({k}_{x}{k}_{z},{k}_{y}^{(2)}{k}_{z},{k}_{z}^{2}-{\varepsilon }_{d}{\mu }_{d}),$$

where ${k}_{y}^{\mathrm{(1)}}={k}_{y}^{\mathrm{(2)}}=\sqrt{{\varepsilon }_{d}{\mu }_{d}{k}_{0}^{2}-{k}_{x}^{2}-{k}_{z}^{2}}$ are the normal (to interface) wave vector components, and the superscripts (1) and (2) refer to two independent polarizations in the dielectric. On the anisotropic medium side ($y < 0$), the eigenfields are given by

39 $${{\bf{H}}}^{(3)}=\frac{1}{{k}_{0}{\eta }_{0}}({k}_{y}^{(3)},-\,{k}_{x},\,0),\,{{\bf{E}}}^{(3)}=-\,\frac{1}{{k}_{0}^{2}{\varepsilon }_{t}}({k}_{x}{k}_{z},{k}_{y}^{(3)}{k}_{z},{k}_{z}^{2}-{\varepsilon }_{t}{\mu }_{t}),$$

40 $${{\bf{H}}}^{(4)}=\frac{1}{{k}_{0}^{2}{\eta }_{0}}({k}_{x}{k}_{z},{k}_{y}^{(4)}{k}_{z},{k}_{z}^{2}-{\varepsilon }_{t}{\mu }_{t}),\,{{\bf{E}}}^{(4)}=\frac{{\mu }_{t}}{{k}_{0}}({k}_{y}^{(4)},-\,{k}_{x},0),$$

where ${k}_{y}^{\mathrm{(3)}}=-\,\sqrt{{\varepsilon }_{z}{\mu }_{t}{k}_{0}^{2}-{k}_{x}^{2}-\frac{{\varepsilon }_{z}}{{\varepsilon }_{t}}{k}_{z}^{2}}$ and ${k}_{y}^{\mathrm{(4)}}=-\,\sqrt{{\varepsilon }_{t}{\mu }_{z}{k}_{0}^{2}-{k}_{x}^{2}-\frac{{\mu }_{z}}{{\mu }_{t}}{k}_{z}^{2}}$, and the superscripts (3) and (4) refer to two independent polarizations in the anisotropic medium. Note that the eigenfields in Eqs (37)–(40) share the common tangential wave vector components *k*_*x*_ and *k*_*z*_ across the interface, as a direct consequence of the phase matching of electromagnetic fields.

According to Maxwell’s boundary conditions, the tangential electric and magnetic field components are continuous at the interface:

41 $${C}_{1}{H}_{x,z}^{\mathrm{(1)}}+{C}_{2}{H}_{x,z}^{\mathrm{(2)}}={C}_{3}{H}_{x,z}^{\mathrm{(3)}}+{C}_{4}{H}_{x,z}^{\mathrm{(4)}},$$

42 $${C}_{1}{E}_{x,z}^{\mathrm{(1)}}+{C}_{2}{E}_{x,z}^{\mathrm{(2)}}={C}_{3}{E}_{x,z}^{\mathrm{(3)}}+{C}_{4}{E}_{x,z}^{\mathrm{(4)}},$$

where *C*_1_, *C*_2_, *C*_3_, and *C*_4_ are constants. The existence of a nontrivial solution of these constants requires that the determinant of the 4 × 4 matrix obtained from Eqs (41) and (42) be zero, which gives the characteristic equation of the surface mode as

43 $$\begin{array}{c}({\varepsilon }_{t}{\mu }_{t}-{\varepsilon }_{d}{\mu }_{d}){k}_{z}^{2}({k}_{x}^{2}+{k}_{z}^{2}-{\varepsilon }_{t}{\mu }_{t}{k}_{0}^{2})-{\varepsilon }_{t}{\mu }_{t}{k}_{0}^{2}({k}_{y}^{(1)}{\varepsilon }_{t}-{k}_{y}^{(4)}{\varepsilon }_{d})({k}_{y}^{(2)}{\mu }_{t}-{k}_{y}^{(3)}{\mu }_{d})\\ \,\,\,+{k}_{z}^{2}[{\varepsilon }_{t}{\mu }_{t}({k}_{y}^{(1)}{k}_{y}^{(2)}+{k}_{y}^{(3)}{k}_{y}^{(4)})-({\varepsilon }_{t}{\mu }_{d}{k}_{y}^{(1)}{k}_{y}^{(3)}+{\varepsilon }_{d}{\mu }_{t}{k}_{y}^{(2)}{k}_{y}^{(4)})]=0.\end{array}$$

### Simulation

The simulation domain is on the *x*-*y* plane and *k*_*z*_ is the out-of-plane wave vector component, which is kept fixed in the simulation so that the eigenwaves possess the same *k*_*z*_^55^. The surface mode is excited by a dipole source placed at the boundary of the metamaterial, which can be implemented by a dipole antenna in the experiments^10,17,35,38^. For the dipole to serve as the source of circular or elliptical polarization, the dipole has two in-plane components and the phase difference in between is set to be *π*/2 or −*π*/2 to mimic the right-handed or left-handed wave^36^.
